# Local experience of referral for breast assessment resulting from incidental findings on CT and PET-CT studies over a 5-year period

**DOI:** 10.1186/bcr3797

**Published:** 2015-11-05

**Authors:** Richard Sidebottom, Jean Lee, Shaheel Bhuva, Vaishali Parulekar

**Affiliations:** 1 Oxford Breast Imaging Centre, Oxford University Hospitals NHS Trust, Oxford, UK

## Introduction

Incidental findings of breast abnormalities from cross-sectional imaging (CT and PET-CT) are a relatively common source of referral for breast assessment at our unit. We sought to describe and quantify our local experience of these referrals and to determine which cross-sectional imaging findings were more predictive of malignancy.

## Methods

Retrospective review using radiology information system searches for mammography referrals resulting from CT and PET-CT scan findings performed over a 5-year period (July 2010−July 2015) in Oxford University Hospitals NHS Trust. Studies in patients with known active breast malignancy were excluded. Cross-sectional imaging characteristics of the abnormalities were collected including CT enhancement, PET avidity, size and shape. Assessment imaging features, subsequent biopsy and clinical outcomes were recorded.

## Results

A total of 126 patients were assessed as a result of incidental breast abnormalities. Thirty-six of 126 (29 %) were subsequently found to have breast malignancy (CT 28/110, 25 % and PET CT 8/16, 50 %). Size, shape and CT enhancement features will be presented. Lesions with high avidity on PET-CT scans were more likely to be primary breast cancer on biopsy (83 % SUVmax >2.5). Of 36 breast malignancies identified, three patients underwent mastectomy surgery, 10 had wide local excision and 20 had non-surgical management. Three patient outcomes are unknown at the time of writing.

## Conclusion

Referrals arising from incidental abnormalities identified on cross-sectional imaging have a high yield for breast malignancy (29 %). Incidental PET findings, while less often a route of referral, have the highest likelihood of identifying a malignant lesion.

